# Indents

**Published:** 1911-02-15

**Authors:** 


### INDENTS.

☞Brief Articles of Technical or Scientific Value will receive notice and comment. Contributions invited.

*The Ordinary Cold.* The common cold is really a catarrh, an inflammation of the upper air passages —down to the trachea or windpipe. Sometimes such a catarrh is purely of a nervous sort—not dependent upon bacteria; such are some cases of hay fever, or the result of some food irritant, or of exposure to dust in certain trades; or one may have a psychic catarrh, merely from the apprehension of taking cold. But in the vast majority of cases colds are the result of infection; and in examining catarrh exudates several germs have been isolated—Friedlander's bacillus; the B. septicus; the B. influenzae; and the micrococcus catarrhalis. As is the case in all infections, there must be two factors to the production of colds; the presence of the essential germ; and the predisposition. And again, as in all infections, individuals differ greatly in susceptibility to catarrh. Many are absolutely immune; they never catch cold, how much soever they may be exposed to the germs. Others show intense susceptibility, blowing their noses incessantly and making themselves and their fellows miserable upon the slightest provocation.

And it is a matter of obvious experience that there are epidemics of colds. An individual will come into a workshop or an office sneezing and snivelling; and presently half the force will be engaged in the like proceeding. It would pay immeasurably to give such a man a day off. The laity generally know the symptoms, which vary slightly according to the parts attacked—the nose, the pharynx or the throat. Nasal obstruction, sneezing, coryza, pain on swallowing, loss of voice, chills and feverish sensations, pains in the bones (from the infection). The common cold is a privilege enjoyed almost wholly by civilized man; it seems to have come in simultaneously with that emblem of civilization, the house. Therein it has gradually become the custom to shut out the fesh air; and with this custom people came to catch cold, a process oftentimes the result of superheating. People who live habitually outdoors know nothing of coughs and colds. Moreover, primitive man was not so overcrowded, nor was he so gregarious as we are; besides, he wore little clothing, such as we wear, to the degree that we become enervated by it; such clothing also harbors germs and carries them from place to place.

Despite our anti-tuberculosis propaganda, which is based largely on pure air, people continue to fear this beneficent agency; the antipathy for it is in principle essentially the same as the tramp evinces for soap and water. In many a rural district the house is hermetically sealed from November to April. Fresh air abounds everywhere except in the houses which man has built; not fresh air, but the want of it, is the cause of many diseases. An ardent Darwinite considers that in the course of time the race will be rendered increasingly resistant to "cold" organisms by the operation of natural selection, since those most resistant to the minor maladies of life (among which are catarrhs) are the most likely to survive and to leave progeny to whom they have transmitted their superior resisting powers. Until that evolutionary era has come to pass, however, we have to do what we can to prevent colds. To this end any local abnormality or affection should be removed or cured—adenoids, enlarged tonsils, spurs or hypertrophied conditions of the nose. Then the general health should be improved; this is another way of saying that predispositions to infection should be eradicated. Stuffy and overheated rooms should be avoided; bedrooms windows should be kept wide open at night. A cool bath (in a warm bathroom) should be habitual, if it can be tolerated. The clothing should be warm, but not heavy. The feet should be invariably warm and dry; "put your chest protectors on your feet."—*Medical Times.*

*Business Axioms.* I have had the pleasure of starting several young men in the profession. The majority of these are to-day successful practitioners. The following are a few axioms I tried to impress on each of these young men: Above all things, believe in yourself. Do not suffer yourself to be led, but try to lead in your profession. Never permit your work to go at a discount; no man is infallible, and if your work should prove defective do it over and over again until you are satisfied it is right. Charge one hundred cents on the dollar for your services. Be sure that your office is a fit place for business, and, above all, make it appear so to your patients.

There is no greater handicap to a dentist than a dingy unkept office; it is a knockdown argument against a man that nothing can overcome; the laity instinctively judge an artist by the point on his pencil.

Be sure to save a little money for the proverbial rainy day, for "into each life some rain must fall," and a time will come when your eyes are not so good and your legs not so strong, your hand not so steady, still the rent collector will come around with his usual regularity.

As to location, I have always favored over the large cities the towns where it is possible for a man to own his own place of business and his own home, where he can become a factor in affairs; if one has the stuff in him to acquire and hold a "swell" city practice he will have the power in a small town, where expenses are less, to draw the most desirable patients from a large area.

As to the question, what would I do different from what I have done? will say that I would be careful of my side issues. The old saying that "Experience teaches a dear school, but fools will attend no other," is very applicable in my case, as I have gone against everything in the calendar, from watered stock to live stock, and I can truthfully say that loaning money and investing in property are the only sane side lines for a dentist. If with any of the others the balance sheet is kept looking right, one hundred chances to one more nervous force has been used up than would have been consumed in that much dental work. Let me enjoin all young men to be suspicious of all stocks and bonds. There are many companies formed to catch dentists and physicians exclusively; their literature is never sent to anyone else. Only recently a man called on me with a scheme to "do" dentists, with "millions in it;" said he was going to let me in on the ground floor, as my acquaintance was more or less extensive in the profession. Among the things he said to tempt me to join him was, "It is a well-known fact among promoters that dentists are the easiest marks of any of the professions, and when one of them fails to bite it is sure thing that some other confidence man has beat you to him."

My advice to all young practitioners is that they join their State and local dental societies. The State society to the other professions is a thing apart, but to dentistry it is the "whole existence." The society is the clearing house of the profession; if they were discontinued the colleges, dental dealers and the public would miss them as much as the dentists themselves, the effect is so far-reaching.—Dr. O. H. Simpson in *Brief*.

*Lactic Ferments in the Treatment of Pyorrhea and other Lesions of the Mouth.* The lactic ferments have the property of proliferating and growing actively, even when the amount of lactic acid which they produce as a by-product reaches 2 and even 3 per cent, where as practically none of the bacteria which infest the orifices of the body, and especially those of the mouth, such as the Klebs Loeffler bacillus, the spirillum of Vincent's Angina, and all of the spirochaetas and micro-organisms which infest the mouth of patients suffering from pyorrhea, are able to withstand even small doses of lactic acid.

The lactic ferments will proliferate and find a means of continuing their life processes by feeding, as it were, on the exudates produced by irritation of other micro-organisms around the roots of the teeth, so that in a short time, when solutions or suspensions of these lactic ferments are injected into the pockets around loose teeth, they produce *in situ* sufficient lactic acid to arrest further developments of objectionable micro-organisms and resist for some time at least, themselves.

With regard to the making of the milk cultures for use in injection into the pockets in pyorrhea, etc., or for a mouth wash, while not of absolute importance, in order to get the best results, the cultures should be made with milk that has been separated from the cream in a centrifugal method, using therefore only skimmed milk, with as little fat as possible.

This milk should then be sterilized in an autoclave at a temperature of 120 degrees C. for twenty minutes and the temperature allowed to fall to 55 degrees C., the container all this time being properly kept from contaimination by the air, by a plug of cotton, as is used in the method employed by bacteriologists. A quart may be inoculated by one or two or three crushed tablets of Fermenlactyl and then placed in the incubator, with precautions taken so that the temperature will not vary more than a few degrees between 50—55 degrees C. for from twenty-four to thirty-six hours, the temperature being maintained as stated during the whole of this time. This will insure very rich cultures of the streptoccoccus and the strepto-bacillus lebeni without any possible contamination of other spore-bearing or other bacteria. It is therefore a pure culture, and such a culture will retain its properties without change for at least seven or eight days if kept in an icebox.

These technical precautions, while not absolutely essential to good dental work, are described simply to show what can be done under the most favorable conditions, but the great predominance of the useful lactic ferments mentioned will prevent proliferation of saprophytic or pathologic micro-organisms to such an extent, that even when the technical details referred to are not strictly observed, such a mouth wash will give first class results.

In order to make this mouth wash of sour milk more easy to handle, the addition of 5 percent. of sugar of milk and 1 per cent. of tragicanth gum, powdered, will aid in suspending the coagulated casein and make it more palatable and agreeable for patients use. This is very easy to obtain and it will suffice to add to the finished product by agitating immediately. Close the recipient and when about to be used, agitate again before using.

At the New York Hospital House of Relief branch, a preparation of this kind is being used under the name of "injection viridi," so that patients may not know exactly what they are using. It is called "injection viridi" because a trace of vegetable chlorophyll is added to give it a pale green color.

During the treatment locally, as shown in my clinic at Asbury Park, I also put the patient on a diet of buttermilk, thus cleansing the stomach and intestinal tract of all fermentative agents. For much clinical date as to the action of these ferments, I am indebted to Dr. Frederick Mason. An exhaustive article could be written, but as this is to serve as a description of a clinic, I will close by answering a few of the questions asked me.

*What is the use of lactic ferments in gouty diathesis*? Gouty manifestations are symptoms of faulty metabolism. Lactic ferments are beneficial in such cases inasmuch as they diminish faulty digestive processes; they prevent formation of endotoxines, diminish the indican output which irritate the kidney, skin and liver and give rise to the deposits within the tissues which give rise to gouty symptoms. Lactic ferments are, therefore, favorable to such cases and cannot do harm.

*What is the effect in diabetes*? While claims have been made in their favor, they have not been proved, and the matter is still under discussion. Lactic ferments cannot harm diabetics in any case. The fact that the lactic acid produced within the intestine is dissipated almost immediately as water and carbonic acid, and instead of rendering the urine more acid, as do inorganic acids, it renders it more alkaline, *i. e.*, less acid, as is well known, is favorable to diabetics.

*What is the effect in anaemia*? In my opinion, lactic ferments would not affect anaemic patients in any way, favorably or otherwise. The cause of the anaemia must be sought and treated accordingly, and the lactic ferment therapy need not be suspended of necessity; in fact, anaemia, in many cases, is due to forms of indigestion When the anaemia is due to gastro-intestinal sepsis (indigestion with formation of foul-smelling gases, etc.), lactic ferments with phenol-phthaleine, would be distinctly indicated. All depends on the cause of the anaemia or choro-anaemia.—Dr. L. A. O'Brien in Dec. 1910 *Items*.

*Conclusion.* To sum it all up, unless the operator can see the end from the beginning, and have the work clearly formulated, at least mentally, he had better spend more time in studying his case before he does anything, and so avoid the necessity of doing the same work twice—*Dental Cosmos.*

*Pressure Anesthesia in Pulp Extraction.* Some practitioners never use pressure anesthesia in this operation; others claim to use it exclusively. Both err somewhat.

Like all other dental operations, many repetitions of the process are necessary to make a dentist able to attain real efficiency in handling cases where pressure anesthesia is indicated for pulp removal. This fact creates the two classes mentioned.

Some practitioners will endeavor to use the method, but being inexperienced—or possibly because they strike a few obstinate cases—they fail to get desirable results in the beginning, become discouraged and fall back into the arsenic rut.

The others meet favorable cases in the beginning; early success makes them enthusiasts for the new process and their enthusiasm sometimes leads to the exclusive use of pressure anesthesia. But there is a happy medium for the use of this process, which some experience and the ever necessary "horse sense" ought to show to any practitioner.

In any case, where decay has progressed so far that its removal means exposure of the pulp, you can use pressure anesthesia satisfactorily. And in those cases where the pulp is almost exposed the method may be employed with nearly the same degree of satisfaction.

It might be made a rule that your degree of ease and success in the use of pressure anesthesia is in proportion to the degree of the advancement of decay toward the pulp. However no rules—only experience—can teach us how and when to proceed with this method. Briefly, this outline of procedure will apply to most cases:

Remove the frail overhanging walls of enamel to gain as free access to the cavity as is possible without cutting into the dentine.

Next, with sharp spoon or battleax excavators lift up and peel out the leathery layers of decay. When skillfully managed, this part of the operation is almost painless. Remember always to exert your force in a direction away from the point of probable exposure and avoid also pressure in the lines of junction between enamel and dentine.

Often an actual exposure is made painlessly in this process. If not and further excavating causes unnecessary torture to the patient, make the exposure with a small, sharp, rapidly revolving bur. That sentence may sound like torture, but the pain caused is only for a second and even the most nervous patients will stand it bravely, if forewarned.

Making the exposure probably starts a flow of blood which is a good thing, especially if your case is a badly congested pulp, or one where a trace of pus is already present and decomposition about to take place.

Next cleanse the cavity with warm water or hydrogen-peroxid and dry it with cotton. Then place the cocain solution in the cavity followed with a piece of unvulcanized rubber of size suited to the cavity; forewarn the patient that a moment's sharp pain will be felt, then proceed to make pressure toward the exposure with any suitable instrument, probably a broad-ended amalgam plugger.

Operators differ as to the form of cocain to use. I have never had better results than with the use of the little pellets of pure cocain hydrochlorate put up by several firms of manufacturing chemists. In this form the cocain is easy to handle, you know exactly how much you are using (each pellet contains 1-12 grain and one-half of a pellet or 1-24 grain is sufficient for most cases) and you can place it exactly where it is wanted, right over the exposure.

For some time I tried moistening the cavity then carrying the cocain pellet with pliers into the cavity; then I tried carrying the pellet of cocain with a moistened pellet of cotton; but the best method of all is to dry the cavity walls with cotton; with a flat-bladed instrument like a battleax No. 19 excavator dipped into water and thus carrying a small drop, touch the cocain pellet and the moisture will enable one to thus pick up the pellet and carry and place it exactly where wanted.

When the pulp is cocainized, the pulp chamber may be opened wide so as to gain free access to each canal and the pulp removed at once.—Dr. R. R. Gillis in *The Summary*.

*Points of Interest for the Busy Dentist.* When busy at the chair the dentist does not have much time for the laboratory. In consequence, he must take a short cut to complete what he does do, and do it well, if there is but a short time in which to complete the work for the patient, otherwise, he can send same to the laboratory expert, who likewise can be benefited by this clinic given.

The points to be brought out are as follows:

(1) A thin solution of red vulcanit rubber dissolved in carbon disulphide. (Do not use any chloroform or gasoline in connection with this solution, but the carbon disulphide instead.) The above solution makes a perfect rubber cement within itself.

(2) In packing a full or partial set of teeth, after the wax is scalded from mold, paint all over with the rubber cement, where you will want to pack the vulcanite rubber. This will retain the rubber where packed, without warming the molds. This is a time-saver over the old way of heating the molds, with the risk of overheating and blowing up the same. Do not apply the rubber cement on a hot mold for it is very volatile and burns on too hot a surface. It will work on a cold mold as well as on a warm one. The rubber cement causes a closer union between the teeth and the vulcanite when used. In applying this cement use a mucilage brush or a small stiff brush which is kept in the bottle of cement, well corked, with a snap or string over the cork to keep it from being forced out, and letting the carbon disulphide evaporate.

(5) The rubber cement is a great time-saver in vulcanite repair work. Take a cracked or broken plate, mount on plaster, and after hardened, remove the plate and cut the broken edges at 45 degrees with a Hall abrasive wheel or a file. Then place back on model; paint with the cement, cut the amount of rubber needed for the occasion, and warm over dry heat until well warmed through, holding the parts of the old plate with one hand, and pressing the new rubber into place firmly with the finger or thumb of the other, then with a sharp or thin instrument, well heated, trim the rubber down to the thickness and form desired. Then invest in the flask, and immediately screw together, and as soon as plaster is hard enough for vulcanizing, proceed to vulcanize. You will find that the repaired plate will come out first-class with a perfect union of the old and new rubbers, without the slightest effort to undercut, which is unnecessary. Pink rubber can be used in connection with the red rubber cement without showing but very little, if any. To clean the old rubber and teeth, use the carbon disulphide, and not chloroform or gasoline.

To replace a very much broken up old plate, wax same together, build up, or make any changes desired, with wax, on the old plate. Invest the same as when teeth are set up on wax. Place in the vulcanizer and run up to the vulcanizing point, then immediately let out the steam and proceed to open up the flask, and pull out the old rubber quickly from the old teeth. (Do all this with leather gloves on, so as not to burn the hands.) If any of the teeth are displaced by removing the old rubber, they can be replaced the same as when using wax. From this point on, proceed the same as when packing a new set of teeth.—Dr. W. H. Upjohn in *Summary*.

*Obtunding Sensitive Dentine.* Apply rubber dam, dehydrate with alcohol and warm air, then apply 50 per cent solution of aromatic sulphuric acid on pledget of cotton, dry cavity with blast of warm air, repeat same till cavity can be excavated without pain. The alcohol and acid should be warmed.—Dr. C. E. White in *Summary*.

*Cotton and Sandarach Varnish Seal Condemned.* I note in "Practical Points," November issue of the *Brief*, an item suggesting cotton and sandarach varnish as a seal over devitalizing applications. I had believed it had long since ceased to be used, and that the advantage of hermetic sealing for that specific treatment was generally appreciated. It has been my experience that when the cavity is well dryed, and the arsenical application is sealed so that moisture and air are positively excluded, there will seldom be any pain worth noticing. Whenever I have used cotton and sandarach it has been followed by severe pain almost invariably. It may be that my formula for devitalizing paste has contributed to the comfortable action under hermetic sealing. It is arsenic and cocaine equal parts by weight, and beechwood creosote q. s.—Dr. C. D. Cheney in *Brief*.

*Comparative Efficiency of some Common Germicides.* There have been so many conflicting statements as to the real efficiency of germicides, especially those in common use for treatment of pathological conditions and instrument and hand disinfection, that the average professional man uses the solution not with a feeling of assurance, but rather of doubt, and trusts to good fortune for satisfactory results.

Bichlorid of mercury has been looked upon as a sort of standard with which other germicides are compared, but from recent tests by Drs. Post and Nicoll, of Chicago, it is shown that bichlorid is not as effective in a one, ten, or thirty-minute exposure, even in as strong a solution as 1-500, as a number of the common germicides, iodin, phenol, trikresol, alcohol, etc.

The tests were thoroughly conducted and necessitated nearly 2,000 inoculations.

The media employed was blood-agar and the pathogenic germs used were streptococci, pneumococci, gonococci and typhoid bacilli.

Bichlorid solutions were found to be slow in their action. Organisms immersed in a 1 to 500 solution were still living in large numbers at the end of ten minutes and one colony of gonococcus was obtained after thirty minutes' exposure. In a 1-2000 solution there was no apparent effect in thirty minutes, but after twenty hours there was complete disinfection. These findings prompted the investigators to say that "contrary to a prevalent opinion the solutions of bichlorid of mercury are absolutely ineffective where prompt disinfection is required, as in the disinfection of hands and fields of operation.

Silver nitrate, even in a 1-5000 dilution was found more effective than a 50 per cent. solution of argyrol or a 10 per cent. solution of protargol.

In the phenol preparations tested Kreso and Trikresol in a 1 per cent. solution killed all organisms in less than one minute. Creolin in 1 per cent. solution and Lysol in 1.5 per cent. were less effective, as was phenol in a less than 5 per cent. solution. A 5 per cent. solution of phenol, however, killed all organisms in less than one minute.

The solutions of Iodin were surprisingly effective. Senn's solution: I 1 part, KI 1 part and aqua 100 parts, and the same with 400 parts of water, and the tincture of Iodin, each killed all organisms in less than one minute.

Liquor Formaldehyde gave unexpected results on account of its slowness of action in solution as strong as 1 per cent., which did not competely kill the germs in thirty minutes.

And a 2 per cent. formalin in glycerin acted more slowly than 1 per cent. in aqueous solution.

Thiersch's solution proves to be a fairly good germicide.

Potassium permanganate was only mildly active in the dilutions used, but even a 1-4000 solution killed the organisms fully as rapidly as a 50 per cent solution of argyrol.

Cupric sulphate showed a distinct effect after thirty minutes, and killed entirely in less than twenty hours.

Boric acid in saturated solution was only slightly germicidal after twenty hours.

Glycerin showed almost no germicidal power.

On the other hand, alcohol, proved to be surprisingly effective. A 20 per cent. solution killed all gonococci in less than ten minutes. With exception of one single colony of pneumococcus, cultures from 30 per cent. alcohol were entirely negative after thirty minutes. Alcohol in 50 per cent. and 70 per cent. solution killed all organism in less than one minute.

The tincture of green soap showed a high germicidal power, killing all organisms in less than one minute.

This is important to dentists for hand and instrument cleansing and disinfection is a subject all are interested in.

In this connection I might add that Dr. H. Selter in *Deut. Med. Wochensch.* states that the use of alcohol is the most appropriate means of rational disinfection of the hands. Its efficiency can be increased by combining alcohol with soap, whereby it is enabled to penetrate into the deeper cutaneous layers and destroy bacteria present there. A suitable alcoholic paste can be made by mixing 86 parts of absolute alcohol and 14 parts of soap. The paste is rubbed into the skin of the hands in amounts of five grams for about five minutes.—Dr. Bethel in *Summary*.

*Lingual Surface of a Vulcanite Denture.* In an article on constructing vulcanite dentures in *Le Laboratoire*, July 18, 1909, page 538, Mr. G. W. Rose suggests that an upper vulcanite denture will feel more natural to the tongue, and be more practical and useful, if the lingual surface is made to resemble that of the mouth with all the natural teeth in place. The conventional form a smooth, even, polished surface, he contends, does not permit the tongue to properly perform its functions, either in speech or mastication. He recommends reproducing the ruga, on the trial plate, and, when preparing the case for flasking carving the lingual surface of the wax supporting the teeth to the form of the natural teeth. This will give much more room for the tongue, and the lingual surface thus formed enables the tongue to assist in the function of speech and mastication to a much greater extent than when this surface is made smooth and even. To overcome the difficulty of polishing this irregular surface he makes moulds of "stent" (a modeling compound much used in Europe), by means of which he stamps a piece of thin, soft metal, for instance, thich tin-foil, to accurately fit the carved wax surface. This is made to extend over the front teeth to a little beyond where the vulcanite will be, and partly over the occlusal surfaces of the bicuspids and molars. This is placed in position when the denture is flasked, and forms part of the mould into which the rubber is packed. If this is neatly made, and nicely cleaned and polished just prior to packing, it produecs on the vulcanite a derse, hard, polished surface that needs no after finish.—*The Dental Brief.*

